# Supplementary material for: The interactome of intact mitochondria by cross-linking mass spectrometry provides evidence for coexisting respiratory supercomplexes
Source: Mol Cell Proteomics. 2017 Dec 8;17(2):216–32. doi: 10.1074/mcp.RA117.000470 (PMC5795388; doi:10.1074/mcp.RA117.000470)
Supplement: Supplemental Data [file supp_RA117.000470_133922_0_supp_23978_fzffwf.zip › spectra_annotation/mito_DR_spectra_annotation/391-1-17-1-15-1.pdf]

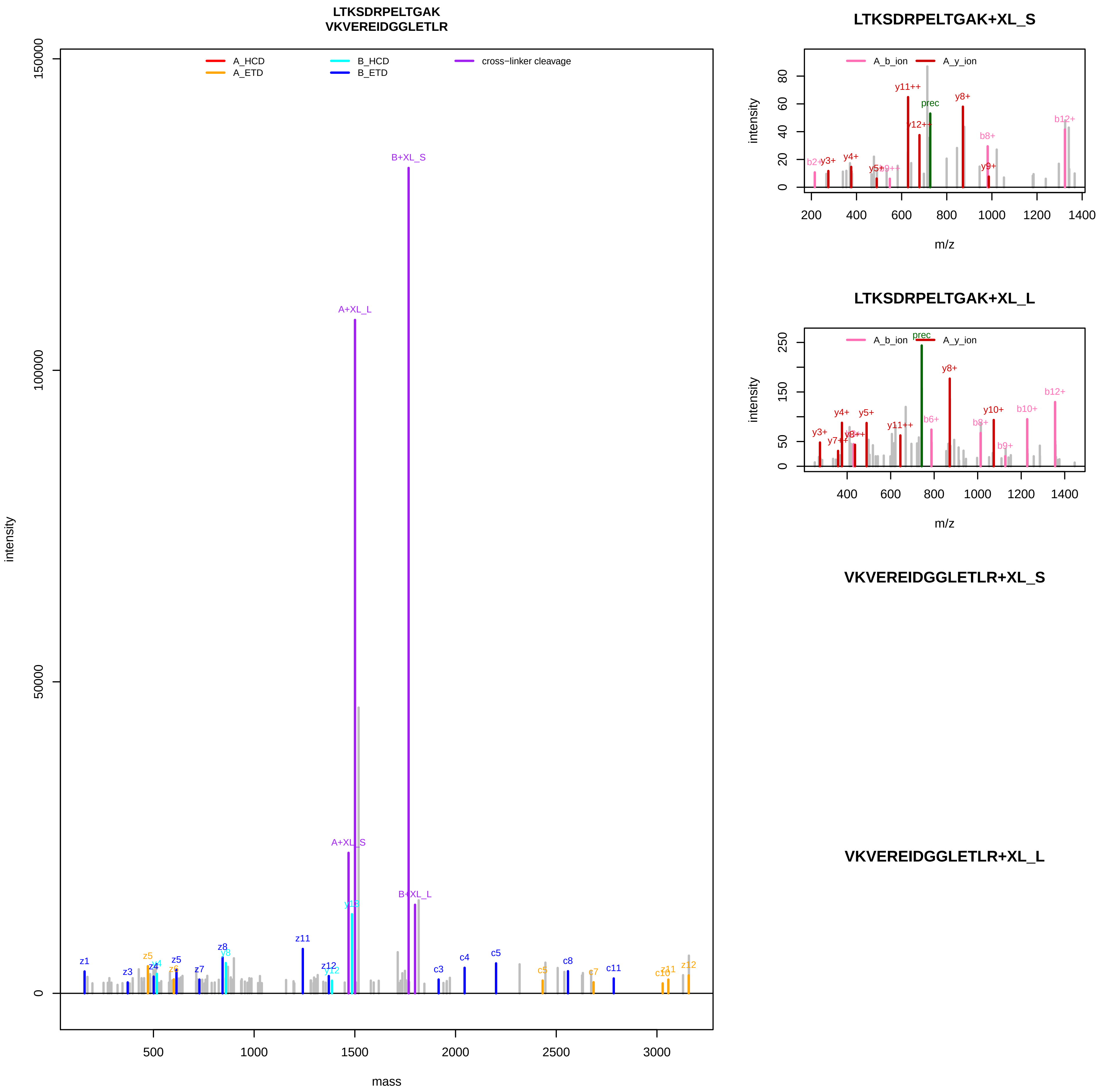
LTKSDRPELTGAK
VKVEREIDGGLETLR
A_HCD
A_ETD
B_HCD
B_ETD
cross−linker cleavage
B+XL_S
A+XL_L
A+XL_S
B+XL_L
intensity
mass
0
50000
100000
150000
500
1000
1500
2000
2500
3000
z1
z3
z4
z5
z7
z8
z11
z12
c3
c4
c5
c8
c11
c5
c7
c10
z11
z12
y13
LTKSDRPELTGAK+XL_S
A_b_ion
A_y_ion
prec
y11++
y12++
y8+
b8+
b12+
y9+
y3+
y4+
y5+
b2+
m/z
LTKSDRPELTGAK+XL_L
y3+
y4+
y5+
y7++
y8++
y11++
b6+
y8+
b8+
y10+
b10+
b9+
b12+
VKVEREIDGGLETLR+XL_S
VKVEREIDGGLETLR+XL_L
